# T‐Cell‐Dependent Bispecific IgGs Protect Aged Mice From Lethal SARS‐CoV‐2 Infection

**DOI:** 10.1002/advs.202406980

**Published:** 2025-02-20

**Authors:** Wenyan Fu, Wei Zhang, Zhongshuai You, Guangyao Li, Chuqi Wang, Changhai Lei, Jian Zhao, Jin Hou, Shi Hu

**Affiliations:** ^1^ Department of Assisted Reproduction Shanghai Ninth People's Hospital Shanghai Jiao Tong University School of Medicine Shanghai 200011 China; ^2^ Department of Biomedical Engineering College of Basic Medical Sciences Second Military Medical University Shanghai 200433 China; ^3^ Department of Respiratory and Critical Care Medicine First Affiliated Hospital Second Military Medical University Shanghai 200433 China; ^4^ Center of Critical Care Medicine First Affiliated Hospital the Second Military Medical University Shanghai 200433 China; ^5^ Department of Biophysics College of Basic Medical, Sciences Second Military Medical University Shanghai 200433 China; ^6^ KOCHKOR Biotech, Inc. Shanghai 201406 China; ^7^ National Key Laboratory of Medical Immunology and Institute of Immunology Second Military Medical University Shanghai 200433 China

**Keywords:** COVID19, SARS‐CoV‐2, neutralization, T cell engagers, variants of concerns

## Abstract

T‐cell ageing may be a key factor in the disproportionate severity of coronavirus disease 2019 (COVID‐19) in older populations. For hospitalized COVID‐19 patients, treatment involving the use of monoclonal antibodies with the ability to neutralize SARS‐CoV‐2 usually involves the administration of high doses but has not been very effective at preventing complications or fatality, highlighting the need for additional research into anti‐SARS‐CoV‐2 therapies, particularly for older populations. In this study, it is discovered that older persons with a severe SARS‐CoV‐2 infection has weaker T‐cell responses. Therefore the development and characterization of spike‐targeting T‐cell‐dependent bispecific (TDB) full‐length human immunoglobulin Gs with enhanced efficacy in the treatment of COVID‐19 is described. Using S‐targeting TDBs, polyclonal T cells are guided to target and destroy S‐expressing cells, preventing the cell‐to‐cell transmission of SARS‐CoV‐2 and thereby eliminating the need for SARS‐CoV‐2‐specific immunity. Using animal models of COVID‐19, it is shown that the selective activation of T cells improves the efficiency of treatment in preinfected mice by attenuating disease‐induced weight loss and death. The significance of T‐cell‐based immunity during infection is highlighted by the findings. These results have implications for better clinical effectiveness of therapies for COVID‐19 and the development of T‐cell‐dependent medicines for the elderly population.

## Graphical abstract

This study describes the development of spike‐targeting T‐cell‐dependent bispecific full‐length human immunoglobulin Gs with enhanced efficacy in the treatment of COVID‐19. The significance of T‐cell‐based immunity during infection is highlighted. These results have significant implications for better clinical effectiveness of therapies for COVID‐19 and the development of T‐cell‐dependent medicines for the elderly population.

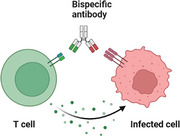

## 1 Introduction

The human population has been impacted by three coronaviruses since 2002, with SARS‐CoV‐2 being the latest. It is the virus responsible for the widespread outbreak of the coronavirus disease known as coronavirus disease 2019 (COVID‐19), which occurred in 2019.^[^ ^1^ ^]^ Casirivimab + imdevimab and bamlanivimab + etesevimab are both combinations of monoclonal antibodies that have received approval from the United States Food and Drug Administration for use in treating patients who have mild‐to‐moderate COVID‐19.^[^ ^2^ ^]^ These monoclonal antibodies neutralize the spike protein of SARS‐CoV‐2. Some studies have revealed that neutralizing monoclonal antibodies, including those that are still awaiting regulatory approval, have shown clear therapeutic benefits in treating mild to moderate COVID‐19 in phase II/III trials. Specifically, they have reduced the risk of hospitalization by more than 80%.^[^ ^3^, ^4^, ^5^ ^]^ The outcomes of phase III trials (ACTIV‐3 and NCT04501978) that have examined the efficacy of these monoclonal antibodies for COVID‐19 patients in hospitals contradict the results of this research, which has produced results that are in direct opposition to those findings. Even when administered at extremely high dosages or in conjunction with remdesivir,^[^ ^6^ ^]^ none of the monoclonal antibodies that were examined showed any therapeutic effect on all patients.

The majority of research into potential SARS‐CoV‐2 treatments has focused on measuring serum spike‐binding or neutralizing antibody titres.^[^ ^7^, ^8^ ^]^ While protection against infection is strongly correlated with the neutralizing activity of an antibody, non‐neutralizing effects are also expected to play roles in attenuating severe sickness and eradicating infection via ancillary immunological mechanisms.^[^ ^9^, ^10^ ^]^ Other functions in addition to neutralization play critical roles in the protection offered by monoclonal antibodies in vivo, as demonstrated by studies involving passive transfer in animal models of COVID‐19.^[^ ^11^, ^12^ ^]^ Moreover, a study revealed that full protection by neutralizing monoclonal antibodies during postexposure therapy necessitates substantial interactions between Fc and FcRs and the activation of monocytes, neutrophils, and natural killer cells.^[^ ^13^ ^]^ The combination of neutralization by antibodies and Fc‐related actions delivers optimal protection, as observed in experiments on animal models of SARS‐CoV‐2. Compared with the original wild‐type antibodies, enhanced variants of monoclonal antibodies with a greater ability to activate Fc receptors have proven to be more effective in these studies.^[^ ^14^ ^]^

Intriguingly, a recent study suggested that SARS‐CoV‐2 can effectively evade host protection, particularly antibody‐mediated responses, by spreading from cell to cell.^[^ ^15^ ^]^ This study contrasted the ability of neutralizing monoclonal antibodies and COVID‐19 convalescent plasma, which are both FDA‐approved for emergency use, to combat SARS‐CoV‐2 infection through cell‒cell transmission and fight against cell‐free infection. Although these therapies are highly effective at preventing SARS‐CoV‐2 infection via cell‐free routes, they have no effect on the ability of the virus to spread from cell to cell. The importance of the ability of the host immune system to directly eliminate virus‐infected cells is beginning to become evident as a result of this work. B cells and T cells constitute the backbone of adaptive immunity. T lymphocytes act as helper cells for other immune cells and can directly kill virus‐infected cells, whereas B cells create antibodies. Additionally, numerous studies have shown that the T‐cell response is an essential component of immunological protection against SARS‐CoV‐2.^[^ ^16^ ^]^ T‐cell responses that are specific for SARS‐CoV‐2 have recently been shown to be important in a number of contexts, including viral clearance, prevention of infection without seroconversion, robust memory, and identification of viral variants. In addition, a recent study revealed that older adults frequently have disorganized adaptive responses, which may be related to a lack of naïve T cells.^[^ ^17^ ^]^ These findings emphasize the importance of the T‐cell response in relation to the severity of disease. Therefore, the use of T cells could increase the antiviral efficacy of therapeutic antibodies, particularly in older individuals.

The creation and development of bispecific molecules that can simultaneously bind T cells and target cells that express antigens, including bispecific T‐cell engager proteins (BiTEs) and CrossMabs, have been aimed primarily at treating cancer.^[^ ^18^, ^19^ ^]^ The binding arms must be engaged at both ends to activate and guide the cytolytic activity of polyclonal T cells in an MHC‐independent manner against antigen‐expressing target cells. The approval of blinatumomab, a bispecific molecule that targets both CD19+ and CD3+ cells, for the treatment of relapsed or refractory B‐cell precursor acute lymphoblastic leukaemia (ALL) shows that these bispecific agents can effectively combat the disease at much lower doses than traditional monoclonal antibodies^[^ ^20^ ^]^ and are both effective and safe in the clinical setting. The ideal treatment development process involves considering the pharmacokinetic (PK) qualities in addition identifying targets. The short half‐lives of blinatumomab and other BiTE and dual‐affinity retargeting (DART) molecules are caused by their lack of Fc domain function, which is responsible for prolonged circulation. Therefore, continuous infusion is required to maintain exposure^[^ ^21^ ^]^ at the same level. Many of these drawbacks might be overcome by a full‐length human IgG1 bispecific antibody that has been designed to increase PK and change Fc‐mediated activities.

In this study, we show that full‐length humanized T‐cell‐dependent bispecific (TDB) therapeutic antibodies are effective against SARS‐CoV‐2‐infected cells using the SPIKExCD3 scenario. The ability of S‐targeting TDBs (S‐TBDs) to recognize infected cells and simultaneously engage receptors on the membrane of polyclonal effector T cells not only neutralizes SARS‐CoV‐2 but also blocks cell‐to‐cell transmission of the virus, avoiding the need to activate preexisting S‐specific cytotoxic effector cells, which overcomes a significant obstacle hindering the efficient elimination of infected cell populations.

## 2 Results

### 2.1 Relationships between Adaptive Immune Responses and COVID‐19 Severity in Older Patients

We set out to measure fundamental metrics of all three arms of antigen‐specific adaptive immune responses to SARS‐CoV‐2 and then relate those antigen‐specific immune responses to COVID‐19 severity in older patients. We measured SARS‐CoV‐2‐specific antibodies (including neutralizing antibodies), SARS‐CoV‐2‐specific CD4^+^ T cells, and SARS‐CoV‐2‐specific CD8^+^ T cells in all individuals in the cohort, with an emphasis on acute cases in elderly patients across two categories of COVID‐19 severity in Changhai Hospital, Shanghai, during the Omicron infections that appeared nationwide in China from December 2022 to February 2023. Seventeen patients with mild disease and 25 patients with severe disease were included in this study (Table S1 and Figure S1A, Supporting Information). The number of days postsymptom onset (PSO) for sample collection ranged from d4 to 33 (Tables S1 and S2, Supporting Information).

**Figure 1:**
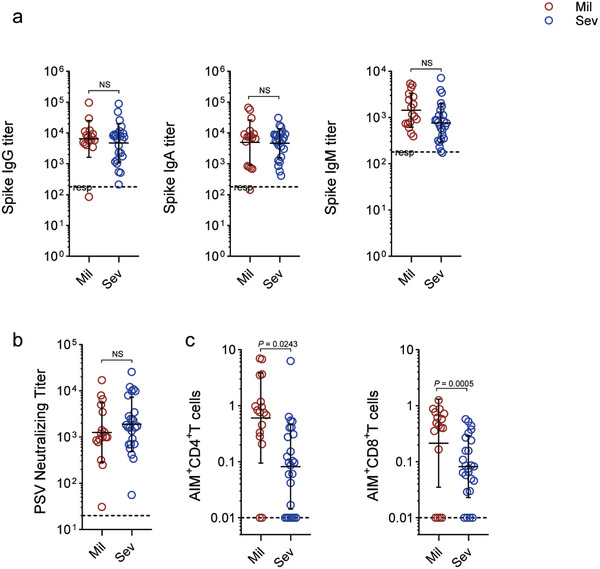
Relationships between adaptive immune responses and COVID‐19 severity. a) Plasma titres of SARS‐CoV‐2 S IgG, IgA, and IgM in patients with mild disease (*n* = 15) and severe disease (*n* = 25). The dotted line indicates limit of detection (LOD). b) Pseudovirus (PSV) neutralizing antibody titres in samples from patients with mild and severe COVID‐19. c) Percentage of background‐subtracted SARS‐CoV‐2‐specific total CD4^+^ and CD8^+^ T cells quantified by AIM after stimulation with spike peptide pools in patients with mild and severe COVID‐19 patients. The percentages indicate the percentage of AIM^+^ T cells after subtraction of the observed background upon DMSO stimulation; the bars indicate the means. The dotted line indicates the responder cut‐off (3 times the limit of detection, LOD) a,b) or LOD c,d). *p* values are from two‐sided nonparametric *t*‐tests.

Total IgG, IgM, and IgA titres against the SARS‐CoV‐2 S protein were determined in every individual. S IgG was detected in every patient (17/17 patients with moderate disease and 25/25 patients with severe disease; **Figure** **1**a), with the exception of one patient whose titres were quite low, which fell within three times the limit of detection (LOD). S IgA (42/42) was also consistently detected. Figure 1a shows that for both mild and severe cases, discernable S IgM was identified less frequently, which aligns with other recent results.^[^ ^22^ ^]^ We utilized neutralization tests to evaluate functional SARS‐CoV‐2 antibodies in addition to measuring antibodies that bind SARS‐CoV‐2. All COVID‐19 patients tested positive for antibodies that neutralize SARS‐CoV‐2 (Figure 1b). In general, the results of these tests revealed that the levels of detectable circulating antibodies against SARS‐CoV‐2 S and neutralizing antibodies were similar in individuals with mild and severe COVID‐19.

Because the T‐cell activation phenotype plays a critical role in the defense against infection, we first tested the activation markers on T cells to investigate the phenotype of T cells. Notably, the frequencies of CD38^+^HLA‐DR^+^CD8^+^T cells in patients with mild symptoms were significantly higher than those with severe symptoms. For CD4^+^ T cells, no statistical differences were observed for frequencies of CD38^+^ HLA‐DR^+^CD4^+^T cells between mild and severe patients (Figure S1, Supporting Information). In addition to evaluating the presence of neutralizing antibodies and T cell activation, we next examined the presence of S‐specific CD4^+^ and CD8^+^ T cells in all participants. PBMCs were stimulated with overlapping peptide pools that corresponded to the full‐length S protein, as shown in Figure 1c. Flow cytometry was used to evaluate the expression of AIM (activation‐induced marker) on CD4^+^ (OX40 and CD137) and CD8^+^ (CD69 and CD137) T cells after stimulation. We identified S‐specific CD4^+^ T cells in 78% of COVID‐19 patients (33 out of 42) based on AIM expression (OX40^+^CD137^+^) upon S‐peptide pool stimulation of the samples. Curiously, a significant decrease in the number of S‐specific CD4^+^ T cells was observed in patients with severe cases compared with patients with mild cases. We detected S‐specific CD8^+^ T cells in 81% of the patients (34 of 42) based on AIM expression (CD69^+^CD137^+^) after S peptide pool stimulation of samples acquired from individuals with COVID‐19. Additionally, these experiments indicated that S‐specific CD8^+^ T‐cell responses were much greater in samples collected from patients with mild cases than in those collected from patients with severe cases.

### 2.2 Development and Characterization of IgG‐Like S/CD3 Bispecific T‐Cell Engagers

**Figure 2:**
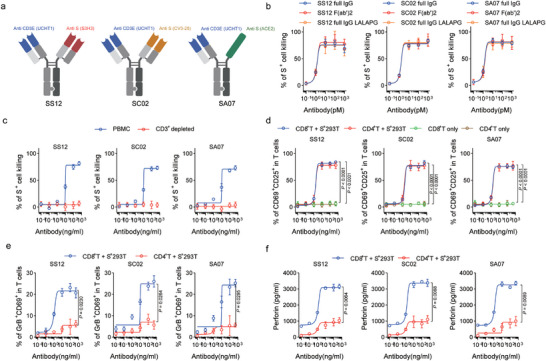
Anti‐S/CD3 TDBs activate T cells in the presence of the target cells and mediates cell killing in a T‐cell‐dependent manner. a) Schematic representation of CrossMabs SS12, SC02, and SA07. b) 293/S cells and PBMCs isolated from healthy donors (1:10 cell ratio) were incubated with various concentrations of full‐length S‐TDBs or F(ab′)2 S‐TDBs for 24 h. c) 293/S cells and PBMCs isolated from healthy donors or PBMCs depleted of CD3^+^ T cells (1:10 cell ratio) were incubated with various concentrations of S‐TDBs for 24 h. d–f) 293/S cells and purified CD8^+^ T cells or CD4^+^ T cells, or CD8^+^ T cells alone were incubated with various concentrations of S‐TDB for 24 h. The T‐cell‐to‐293 cell ratio was 5:1. Cell killing and activated T cells, marked as CD69^+^CD25^+^ cells, were measured and calculated. Granzyme B (GrB) induction was also detected using fluorescence‐activated cell sorting (FACS), and the perforin concentration in the medium was measured with enzyme‐linked immunosorbent assays (ELISAs). The data are presented as the means ± s.d.s b–f). *p* values are from two‐way ANOVAs followed by the Bonferroni post hoc correction d) and two‐sided paired *t*‐tests e,f).

Overall, associations were found between strong SARS‐CoV‐2‐specific T‐cell responses and low COVID‐19 severity. We therefore sought to develop biological agents that have the potential to improve T‐cell immunity. According to the Chinese Center for Disease Control and Prevention,^[^ ^23^ ^]^ the most prevalent SARS‐CoV‐2 lineages were BA.5 and its subvariants in China in December 2022 to Feb 2023. To avoid losing the substantial capacity to combat SARS‐CoV‐2 Omicron subvariants, the anti‐S antibody S3H3,^[^ ^24^ ^]^ the CV3‐25^[^ ^25^ ^]^ antibody, and the ACE2 protein^[^ ^26^ ^]^ were used to generate three anti‐S/CD3 bispecific antibody‐like molecules. These molecules were designed to achieve combined effects and were given the names SS12, SC02, and SA07, respectively. These two antibodies have been proven to bind to conserved regions of the S protein and the ACE2 protein has the potential to combat SARS‐CoV‐2 variants. We did not choose an antibody that binds to the receptor‐binding domain (RBD) or N‐terminal domain (NTD) of the S protein since previous research^[^ ^27^ ^]^ has indicated that these antibodies have lost a substantial amount of their capacity to combat SARS‐CoV‐2 Omicron subvariants. The “knobs into holes” methodology^[^ ^28^ ^]^ and the CrossMab methodology^[^ ^29^ ^]^ were utilized to construct a heterodimerized heavy chain and differentiate between the two light chain/heavy chain interactions (**Figure** **2**a). By introducing the P329G LALA mutation, FcR binding was rendered completely ineffective. The monovalent affinities of the parental and bispecific antibodies for the trimeric S protein and recombinant CD3delta epsilon were determined using surface plasmon resonance (SPR). No discernible differences in the affinities of the bispecific IgGs and the parental antibodies for their respective ligands were observed, and SPR assays showed that the antibodies bound to both ligands simultaneously (Table S3, Supporting Information). The key pharmacokinetic (PK) characteristics of SB12, SC02, and SA07 in mice were extremely comparable to those of S3H3, CV3‐25, and ACE2‐Fc, as described in Table S4 (Supporting Information). This result indicated that SS12, SC02, and SA07 have PK features similar to those of a typical IgG molecule.

### 2.3 S‐TDBs are Conditional T‐Cell Agonists Whose Actions are Contingent on the Presence of Both T Cells and Targets

In the next step, we characterized the mechanism of action for anti‐S/CD3 TBDs. We first verified that functional activity was not dependent on the presence of antibody constant domain sections, even though the molecule was designed to include a heterodimeric Fc region with P329G LALA mutations^[^ ^30^ ^]^ for total inactivation of FcgR and C1q couplings. An in vitro potency assay was performed utilizing healthy human donor peripheral blood mononuclear cells (PBMCs) together with an F(ab′)2 fragment of anti‐S/CD3 IgGs that lacked the Fc region. This study was conducted using the S‐expressing 293T cell line. The fragment exhibited the same molar potency as full anti‐S/CD3 IgGs (Figure 2b), suggesting that Fc receptor‐mediated ADCC does not significantly contribute to the activity of anti‐S/CD3 IgGs. On the other hand, the activity of anti‐S/CD3 IgG was eliminated when CD3‐expressing cells were removed from PBMCs (Figure 2c), demonstrating that T cells are necessary for activity.

Purified CD4^+^ or CD8^+^ T cells were utilized as effector cells, and they were activated by anti‐S/CD3 IgGs with an equivalent level of efficacy, as determined by the stimulation of both CD69 and CD25 expression (Figure 2d). The presence of S^+^ target cells was absolutely necessary for anti‐S/CD3 IgGs to induce T‐cell activation (Figure 2d). Our data further showed that single‐armed binding of parental antibodies to S protein or to CD3 is insufficient to activate T cells (Figure S2, Supporting Information). Our results revealed a noticeable increase in the level of the intracellular protein granzyme B (Figure 2e), which was more pronounced in activated CD8^+^ T cells than in CD4^+^ T cells. Additionally, we detected increased levels of perforin (Figure 2f) released into the cell culture medium in these samples. Anti‐S/CD3 IgGs were further tested in dose‒response assays across a panel of S^+^ cell lines with various mutations derived from SARS‐CoV‐2 Omicron variants of concern (VoCs) and in vitro half‐maximal effective concentration (EC50) values ranging from 0.23 to 5.89 ng mL^−1^ for SS12, 4.43 to 15.05 ng mL^−1^ for SS12, and 1.18 to 7.78 ng for SA07, respectively (Figure S3, Supporting Information). Anti‐S/CD3 IgGs had no effect on 293T cells since these cells do not express the S protein (Figure S3, Supporting Information).

### 2.4 Superior Effect of S‐TDBs on a Cell‐to‐Cell SARS‐CoV‐2 Transmission Model

SARS‐CoV‐2 cell‐to‐cell transmission is the key strategy by which the virus evades host protection, although neutralizing antibodies are efficient in preventing cell‐free infection by the virus. An intron‐Gaussia luciferase (inGluc) HIV‐1 lentiviral vector‐based pseudotyped virus system15 was adopted to investigate the susceptibility of SARS‐CoV‐2 spike‐mediated cell‐to‐cell transmission to neutralization by antibodies directed against the spike protein, ACE2‐Fc fusion protein, and bispecific IgGs in the presence of effector immune cells.

**Figure 3:**
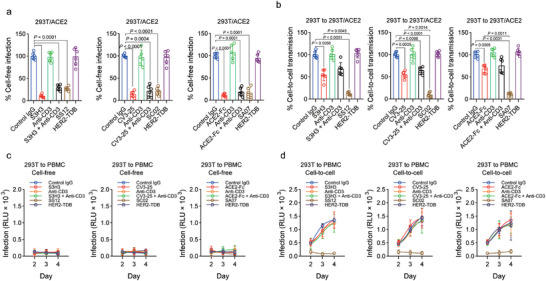
Anti‐S/CD3 TDBs are effective in blocking the cell‐to‐cell transmission of SARS‐CoV‐2. The inGluc‐based lentiviral pseudotypes bearing the spike protein were produced in 293T cells, which were cocultured with target cells (293T/ACE2) and PBMCs for cell‐to‐cell transmission. The Gluc activity of the cocultured cells was measured over time. a) Cell‐free infection was performed in Transwell plates, from which Gluc activity was measured. The indicated therapeutic IgGs were present during the infection period. The results were obtained from six independent experiments (*n* = 6). b) A comparison of cell‐to‐cell transmission mediated by SARS‐CoV‐2 indicated that therapeutic IgGs were present during the infection period. The results shown were obtained from six independent experiments, with cell‐free infection measured at 48 and 72 h after coculture. The portion of cell‐free infection was excluded (*n* = 6). c) A comparison of cell‐free infection mediated by SARS‐CoV‐2 in the presence of the indicated therapeutic IgGs during the infection period. The target cells were human PBMCs (*n* = 6). d) A comparison of cell‐to‐cell transmission mediated by SARS‐CoV‐2 indicated that therapeutic IgGs were present during the infection period. The target cells were human PBMCs alone. The results shown were obtained from six independent experiments, with cell‐free infection measured at 48 and 72 h after coculture. The portion of cell‐free infection was excluded (*n* = 6). The data are presented as the means ± s.d.s; *p* values are from one‐way ANOVA followed by Tukey's post hoc test a,b).

Our research indicated that S‐targeting monospecific IgGs, such as S3H3, CV3‐25, and ACE‐Fc, effectively inhibited cell‐free SARS‐CoV‐2 infection in 293T/ACE2 cells by more than 90% in the presence of PMBCs. On the other hand, combined therapy or bispecific IgGs strongly inhibited cell‐free infection, and they showed similar effect compared with their parental antibodies (**Figure** **3**a). Notably, only S3H3 had a significant inhibitory effect on the cell‐free infection model compared with S3H3 plus anti‐CD3 or SS12 treatment (*p* = 0.0256 and 0.0007). On the other hand, S‐targeting monospecific IgGs substantially decreased cell‐to‐cell transmission between 293T and 293T/ACE2 cells to less than 50% (Figure 3b), and the combination of S‐targeting IgGs with anti‐CD3 antibodies had a similar effect. Bispecific IgGs, but not their parental antibodies, significantly reduced SARS‐CoV‐2 replication following infection in the presence of effector immune cells during cell‐to‐cell transmission but had little impact on cell‐free infection. The depletion of CD3‐expressing cells from peripheral blood mononuclear cells (PBMCs) completely eliminated the activity of bispecific IgGs, confirming the hypothesis that T cells are necessary for the efficient suppression of cell‐to‐cell infection by these antibodies (Figure S4, Supporting Information).

Cell‐to‐cell transmission of SARS‐CoV‐2 has been previously reported to occur in peripheral blood mononuclear cells (PBMCs), which do not express the ACE2 protein.15 In PBMCs, we tested both cell‐to‐cell transmission and cell‐free infection when anti‐S monospecific IgGs or bispecific IgGs were present in the culture medium. Notably, the spread of SARS‐CoV‐2 from one cell to another increased from the second day to the fourth day, and monospecific IgGs targeting S had a negligible effect. Our data revealed that although no or a low level of cell‐free infection was detected in PBMCs (Figure 3c), consistent with recently published results, an increase in cell‐to‐cell transmission was observed for SARS‐CoV‐2. In contrast, no evidence of cell‐to‐cell infection could be observed in PBMCs that had been treated with bispecific antibodies during the course of the 3‐day timeframe. Collectively, our findings showed that T‐cell‐activating bispecific antibodies can prevent the spread of lentiviral pseudotypes harboring the SARS‐CoV‐2 spike protein from cell to cell.

### 2.5 S‐TDBs are Highly Effective in Therapeutic Models of Aged Mice

Because aged mice exhibit increased severity of SARS, we used 12‐month‐old mice in our experiments. Unfortunately, the preclinical characterization of T‐cell‐recruiting bispecific antibodies has been severely hampered by the absence of antibody cross‐reactivity to nonhuman animals.^[^ ^31^ ^]^ The introduction of a transgenic mouse strain expressing human CD3 allowed for in‐depth experiments in a mouse model with a fully functioning immune system. Following the inhalation of a modified adeno‐associated virus (AAV‐hACE2), mice were further humanized with ACE2 in the cells of the upper and lower respiratory tracts.^[^ ^32^, ^33^ ^]^

**Figure 4:**
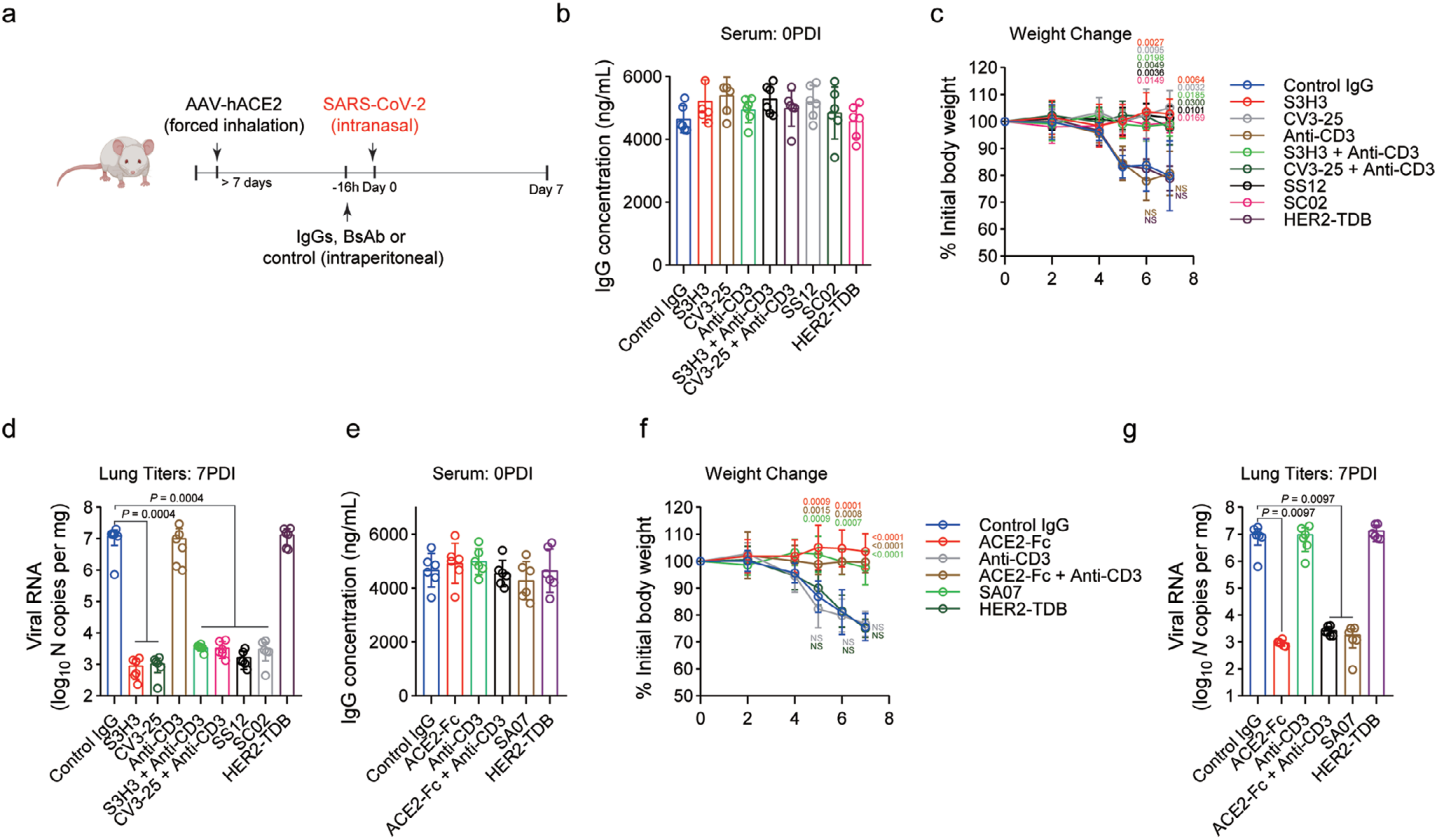
Neutralizing activity is sufficient for the prophylactic efficacy of therapeutic IgGs against SARS‐CoV‐2 infection in AAV‐hACE2 humanized CD3e mice. a) Humanized CD3e mice were transduced with AAV‐hACE2 by forced inhalation. After >7 days, the mice were inoculated intraperitoneally with antibodies (12 mg kg^−1^ for each drug) 1 day before (black arrow) being infected intranasally with SARS‐CoV‐2. b) Serum concentrations (ng mL^−1^) of the indicated IgGs at the time of challenge (0 dpi) (*n* = 8). c) Weight change following the administration of the indicated IgGs (*n* = 7 or 8). d) Viral RNA levels in the lung at 7 dpi (*n* = 7 or 8). e) Serum concentrations (ng mL^−1^) of the indicated IgGs or ACE2 fusions at the time of challenge (0 dpi) (*n* = 8). f) Weight change following the administration of the indicated IgGs (*n* = 7 or 8). g) Viral RNA levels in the lung at 7 dpi (*n* = 7 or 8). The data are presented as the means ± s.d.s b–g), *p* values are from two‐way ANOVA followed by the Bonferroni post hoc correction c,f) compared with the control) and one‐way ANOVA followed by Tukey's post hoc test d).

In prophylaxis investigations, ACE2 humanized CD3 transgenic mice received a single dose of anti‐S monospecific IgGs or S‐TDBs intraperitoneally 16 h before intranasal inoculation with SARS‐CoV‐2 Omicron BA.5 (10^4^ plaque‐forming units [PFUs]) (**Figure** **4**a). The anti‐CD3 antibody, human epidermal growth factor receptor 2 T‐cell binding fragment (HER2‐TDB), and an isotype control IgG were administered as controls. The serum levels of therapeutic IgGs were comparable between the groups at the time of SARS‐CoV‐2 exposure (Figure 4b). The effects on SARS‐CoV‐2‐induced weight loss were comparable between anti‐S monospecific IgGs and S‐TDBs, with a loss of protection observed only in the control groups (Figure 4c), indicating that both monospecific and bispecific antibodies have the capacity to prevent weight loss in this stringent challenge model, and anti‐S monospecific and bispecific antibody treatment significantly reduced the viral burden in the lung at 7 days postinfection (dpi) (Figure 4d). The prophylactic effect of ACE2 fusion proteins was examined in a separate group of mice. Both ACE2‐Fc and SA07 prevented weight loss in the SARS‐CoV‐2 exposure paradigm, and the blood levels of therapeutic IgGs were similar across the groups (Figure 4e,f). At 7 days postinoculation, a substantial reduction in the viral load was observed in these treatment groups (Figure 4g). In our experiments, we did not detect a discernible difference in either the amount of weight lost or the viral load. Consequently, when a highly neutralizing antibody is used for prophylaxis, the T‐cell‐dependent effector actions of bispecific IgGs are unnecessary.

**Figure 5:**
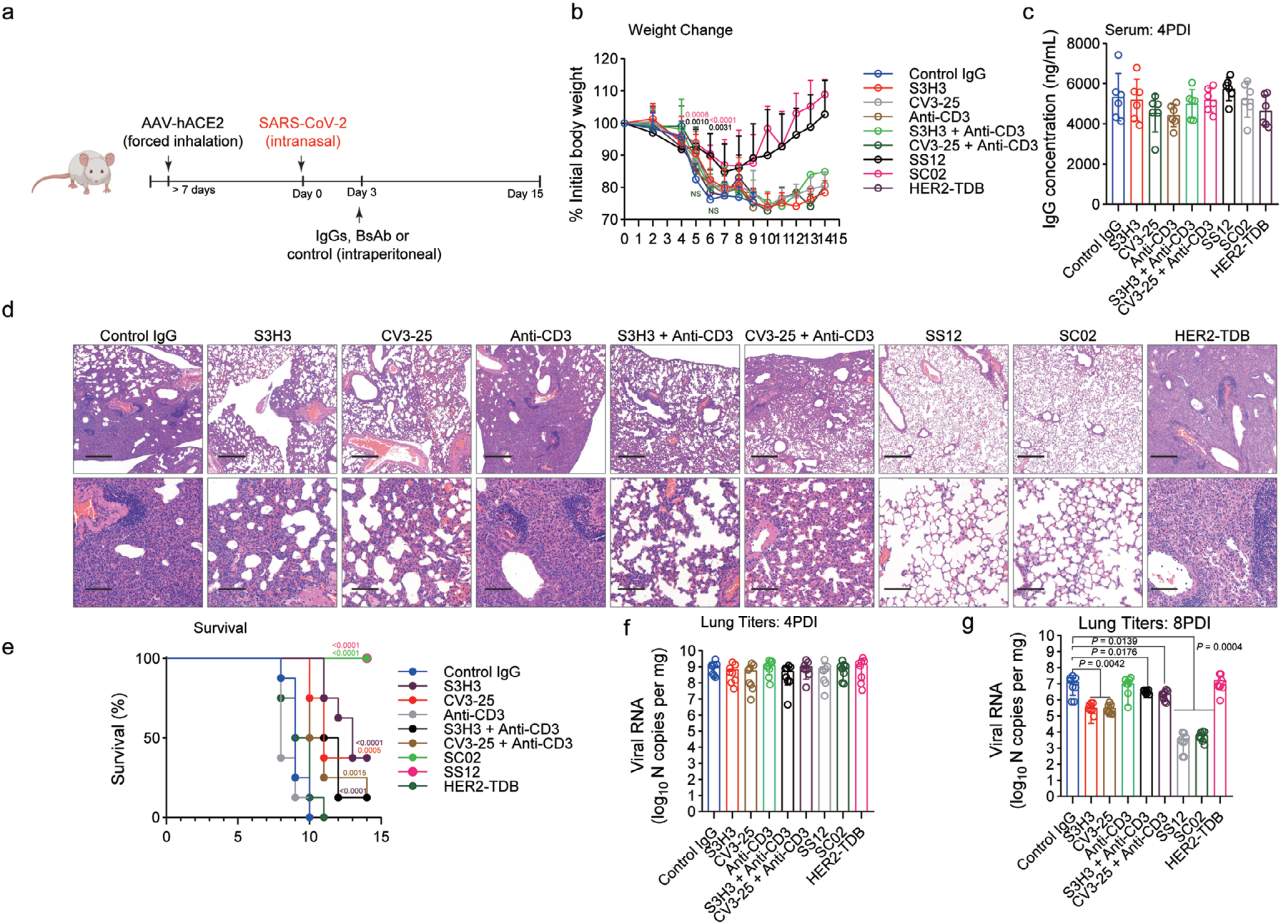
T‐cell effector functions are required for the therapeutic activity of neutralizing anti‐SARS‐CoV‐2 antibodies in mouse infection models. a) Humanized CD3e mice were transduced with AAV‐hACE2 by forced inhalation. After >7 days, the mice were inoculated intraperitoneally with antibodies (12 mg kg^−1^ for each drug) 3 days (black arrow) after being infected intranasally with SARS‐CoV‐2. b) Weight change following the administration of the indicated IgGs (*n* = 7 or 8). c) Serum concentrations (ng mL^−1^) of the indicated IgGs at 4 dpi (*n* = 8). d) Histopathology. Haematoxylin and eosin (H&E)‐stained sections of paraffin‐embedded lungs from infected mice are shown. Images are representative of two separate experiments (*n* = 7 or 8 mice per group). Scale bars, 500 µm (H&E, upper panel), 100 µm (H&E, lower panel). e) Survival analysis (*n* = 8). f,g) Viral RNA levels in the lungs at 4 and 8 dpi (*n* = 7 or 8). The data are presented as the means ± s.d.s, b,c,e–g), and the *p* values are from two‐way ANOVA followed by the Bonferroni post hoc correction (b) compared with the control), log‐rank (Mantel–Cox) test e) and one‐way ANOVA followed by Tukey's post hoc test g).

Although T‐cell effector activity was not needed when S‐TDBs were provided prophylactically, we re‐evaluated this phenomenon in the postexposure context. We used intranasal inoculation to introduce SARS‐CoV‐2 into ACE2‐humanized CD3 transgenic mice, followed by the intraperitoneal administration of a single dose of therapeutic antibodies on Day 3 postinfection (dpi) (**Figure** **5**a; and Figure S5, Supporting Information). Compared with control IgG‐treated animals, mice administered either S3H3 or CV3‐25, which had previously prevented weight loss in a prophylaxis model, lost the protection afforded by the passive transfer of the bispecific antibodies SS12 and SC02 (Figure 5b). Because equal concentrations of IgGs were detected in the serum at 4 dpi, the variations in protection could not have been caused by the IgG concentrations (Figure 5c). The lung pathology of the mice revealed widespread damage to the alveoli, with 50%–80% of the tissue area affected. This damage was accompanied by the infiltration of immune cells and fibroblasts that replaced the alveoli, thickened septa, and the presence of activated macrophages with foamy cytoplasm, which mirrored the findings associated with severe COVID‐19 in humans^[^ ^34^ ^]^ (Figure 5d). The mice treated with SS12 or SC02, on the other hand, not only maintained their body weight but also showed no macroscopic or histological alterations (diffuse alveolar damage of less than 5%–10%) (Figure 5e). Lethality was also decreased by treatment with bispecific antibodies (Figure 5f). At 4 days postinfection, treatment with anti‐S IgGs or bispecific antibodies did not reduce viral RNA levels in the lung when compared to isotype control treatments. However, at 8 days postinfection, treatment with bispecific antibodies, but not with monospecific anti‐S antibodies, reduced the levels of infectious virus in the lungs (Figure 5g). Additionally, mice receiving bispecific antibodies exhibited improved pulmonary mechanics (e.g., inspiratory capacity, resistance, and elastance), but those receiving monospecific anti‐S antibodies did not (Figure S6, Supporting Information). These findings suggest that bispecific antibodies' T‐cell effector capabilities are advantageous for the best therapeutic action to lessen the clinical illness caused by SARS‐CoV‐2 infection.

**Figure 6:**
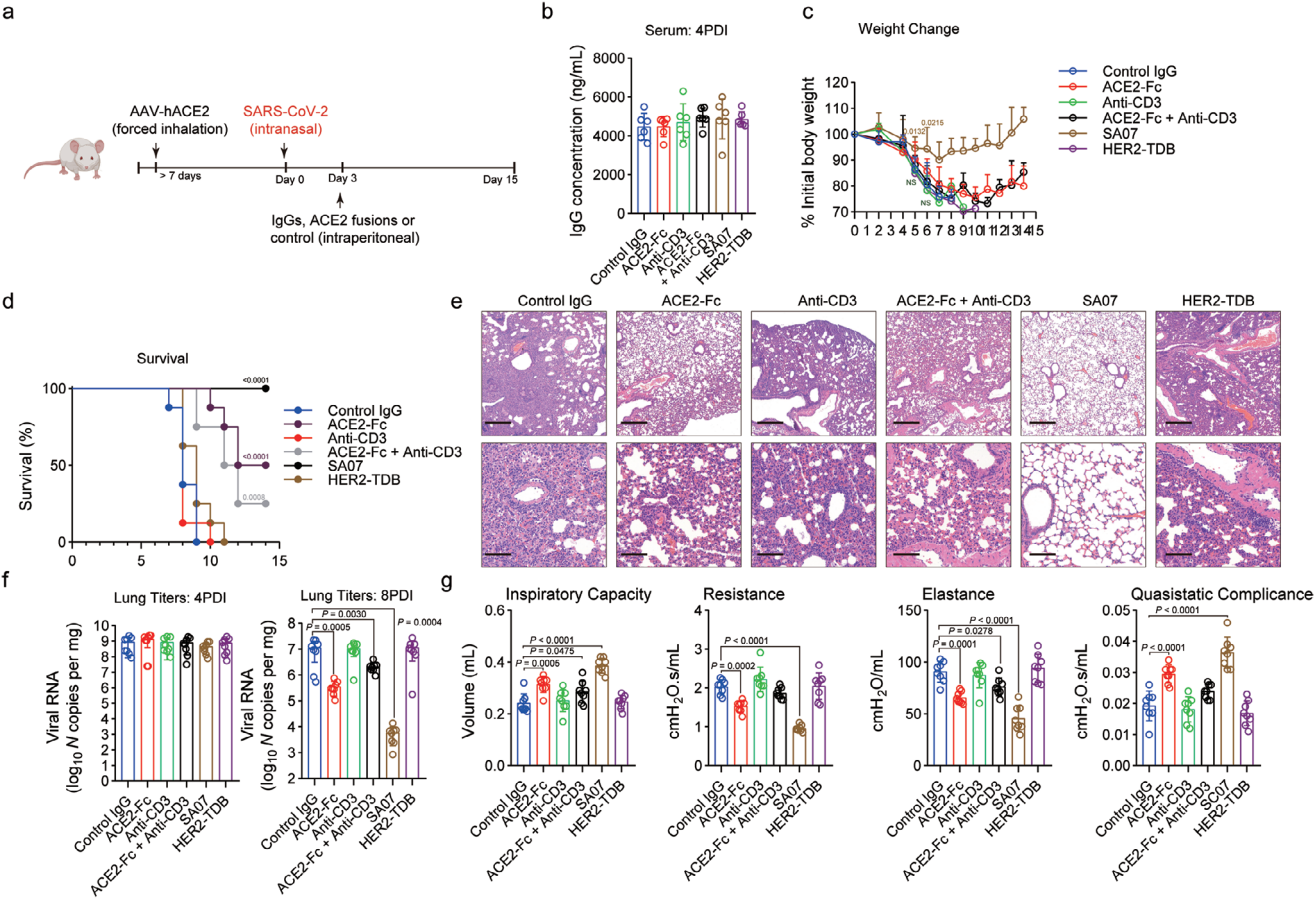
T‐cell effector functions enhance the therapeutic activity of S‐TDB against SARS‐CoV‐2 in mice. a) Humanized CD3e mice were transduced with AAV‐hACE2 by forced inhalation. After >7 days, the mice were inoculated intraperitoneally with different ACE2 fusions or control Abs (12 mg kg^−1^ for each drug) 3 days (black arrow) after being infected intranasally with SARS‐CoV‐2. b) Serum concentrations (ng mL^−1^) of the indicated IgGs at 4 dpi (*n* = 8). c) Weight change following the administration of the indicated IgGs (*n* = 7 or 8). d) Survival analysis (*n* = 8). e) Histopathology. Haematoxylin and eosin (H&E)‐stained paraffin‐embedded lung sections from infected mice are shown. Images are representative of two separate experiments (*n* = 7 or 8 mice per group). Scale bars, 100 µm (H&E, upper panel), 500 µm (H&E, lower panel). f) Viral RNA levels in the lung at 4 and 8 dpi (*n* = 7 or 8). g) Parameters of respiratory mechanics measured at 8 dpi: inspiratory capacity, resistance, elastance, and quasistatic compliance (*n* = 7 or 8). The data are presented as the means ± s.d.s, b,c,f,g), and the *p* values are from two‐way ANOVA followed by the Bonferroni post hoc correction (c) compared with the control), the log‐rank test (Mantel–Cox) d) and one‐way ANOVA followed by Tukey's post hoc test f,g).

Comparable outcomes were observed when we evaluated the therapeutic efficacy of ACE2 fusion proteins in vivo (**Figure** **6**a). At 4 days postinfection, similar levels of IgG were detected in the serum of this group (Figure 6b). Only SA07, but not ACE2‐Fc, prevented disease‐induced weight loss and conferred protective effects when delivered to CD3 transgenic mice (Figure 6c,d). In contrast to the anti‐S bispecific ACE2 fusion, anti‐CD3 antibodies, and HER2‐TDBs did not result in increased therapeutic effectiveness because neither of these IgGs protected mice from lethal SARS‐CoV‐2 exposure (Figure 6d). When administered at 3 days postinfection (dpi), SA07 significantly decreased alveolar damage, lowered SARS‐CoV‐2 viral RNA levels in the lungs by 8 dpi, and improved pulmonary mechanics (Figure 6e,f,g). These results imply that targeted activation of CD3 engagement may enhance the therapeutic effectiveness of neutralizing monoclonal antibodies.

## 3 Discussion

The ageing process poses a significant risk for the progression, severity, and clinical outcome of COVID‐19. The process of T‐cell ageing is crucial in increasing the susceptibility of elderly individuals to infections and reducing their ability to respond effectively to vaccines.^[^ ^35^ ^]^ Here, we present that the spike‐related T‐cell immune response in old individuals was weaker in patients with severe COVID‐19 than in patients with mild cases. We also described the development of bispecific antibodies that may redirect T lymphocytes to SARS‐CoV‐2‐infected cells. These antibodies have biochemical and pharmacological features that are characteristic of full‐length IgGs. The CD3‐based antibody T‐cell redirection engineering does suffer one significant challenge, which is the requirement of a second stimulatory signal to achieve full T‐cell activation and prevent anergy. Multiple bispecific formats have been developed to meet or circumvent this requirement, and only single‐armed binding of bispecific antibodies to CD3 is insufficient to either activate T‐cells^[^ ^36^ ^]^ or to induce anergy^[^ ^37^ ^]^ both in the previous reports and our data. The in vitro S^+^ cell depletion experiments described in this report provide insights into the most important mechanistic features of S‐TDBs. These features include broad activity against cells expressing wild‐type spike or Omicron subvariants, as well as T‐cell‐dependent killing of target cells through the granzyme–perforin pathway. The appearance of SARS‐CoV‐2 VoCs^[^ ^38^ ^]^ has cast doubt on the feasibility of the initial plans to acquire protective immunity to the virus by infection and/or vaccination. The effectiveness of neutralizing antibodies present in the serum of individuals who have received vaccinations and those who have contracted earlier strains of the virus, as well as recombinant neutralizing antibody cocktails, has been studied^[^ ^39^, ^40^ ^]^ and has been reduced as a result of mutations in the SARS‐CoV‐2 spike protein. The latter protein is the primary antigenic target in the majority of approved COVID‐19 vaccines. We focused on neutralizing anti‐SARS‐CoV‐2 monoclonal antibodies that are still effective against VoCs and evaluated their protective effects on mouse strains that replicate human CD3e cells in vivo. This approach allowed us to maximize the translational applicability of our findings. Furthermore, the ACE2 receptor was employed in this research because of its resistance to escape mutations in viruses.

Cell‐free infection with SARS‐CoV‐2 was almost completely blocked by neutralizing anti‐SARS‐CoV‐2 monospecific antibodies and S‐TDBs, but cell‐to‐cell transmission of SARS‐CoV‐2 was, to a large extent, refractory to anti‐SARS‐CoV‐2 monospecific antibodies, as shown by experiments assessing the effects of S‐TDBs on virus transmission. On the other hand, S‐TDBs were effective in both experimental routes employed. We investigated the activity and potency of S‐TDBs in in vivo prophylaxis and therapy models. In these experiments, although T‐cell engagement functions were not required when neutralizing monospecific antibodies, such as S3H3 and CV3‐25, were administered as prophylaxis in mice, functional CD3e activation was favored for optimal protection as postexposure therapy. This increased the therapeutic efficiency of S‐TDBs compared with their parental antibodies and corresponded with a decreased viral burden, improved pulmonary function, decreased inflammatory responses, and sustained tissue repair processes.

We discovered that activated T cells were not necessary when neutralizing monoclonal antibodies were used as a prophylactic measure. This result implies that the effectiveness of neutralizing antibodies in preventing viral exposure is due mainly to their ability to neutralize the virus, thereby preventing the initial viral infection and limiting its spread. This finding is consistent with the in vitro data showing that neutralizing monospecific antibodies are effective at blocking the cell‐free route. This pathway is closely tied to a virus's ability to infect target cells and results in the transmission of the virus from one person to another through direct contact. On the other hand, cell‐to‐cell transmission is a major factor contributing to the progression of diseases caused by viruses.^[^ ^41^ ^]^ Although the Fc effector functions of the neutralizing antibodies help eliminate cells infected with SARS‐CoV‐2 by promoting the presentation of antigens and activating CD8^+^ T‐cell responses that are specific to the virus, this approach was not effective in our mouse model, partly because of the challenge of accelerated weight loss and significant mortality in ACE2‐humanized mice with SARS‐CoV‐2 in a very short time, which resemble the clinical manifestations of severe COVID‐19. The rapid onset of symptoms may limit the generation of a full T‐cell response and prevent a complete dissection of the mechanisms of COVID‐19 pathogenesis and the potential immunopathologies resulting from host immune‐related reactions.

Our study has several limitations. We provide evidence that targeting S protein andCD3 with bispecific antibodies can be achieved, but our in vivo efficacy models may not fully recapitulate human infection. Particularly, since the study of T cell phenotypes in mouse lung injury is relatively complex,^[^ ^42^ ^]^ it is difficult to directly determine the function of bispecific antibodies by simply detecting the phenotypes of lung‐derived T cells. Therefore, detailed exploration and follow‐up research is needed. Moreover, our study did not show direct evidence to that therapeutic efficacy in vivo was attributed to reduce cell to cell transmission of SARS‐CoV‐2, as in vivo experiments showed the comprehensive treatment effects.

Although Bispecific IgG neutralizing antibodies against SARS‐COV‐2 have already been reported,^[^ ^32^, ^43^ ^]^ these bispecific IgGs were constructed based on different S protein targeting antibodies with different epitopes. We developed anti‐CD3–anti‐S bispecific antibodies, which are bispecific T‐cell engager IgGs. These bispecific IgGs bind to a CD3 protein on healthy T cells and, at the same time, to an S protein on infected cells. This brings the T cells and infect cells close together so the T cells can more effectively kill the infected cells. Moreover, therapeutic models of aged mice (12‐month‐old mice) were adopt in our study, give more insight to the mechanism of action of antibodies usage in aged satiation. Neutralizing anti‐SARS‐CoV‐2 monoclonal antibodies are expected to be a key component in measures to control the spread of the virus and treat COVID‐19, especially in older populations or those who are at high risk or have compromised immune systems, in the near future. Multiple studies have reported the development and characterization of neutralizing monoclonal antibodies, the majority of which target the RBD of the SARS‐CoV‐2 spike (S) glycoprotein and inhibit ACE2 binding.^[^ ^44^ ^]^ Our results highlight the potential for the selective activation of T‐cell immunity as a method for developing monoclonal antibody‐based therapies with increased potency and improved therapeutic efficacy against COVID‐19, especially in the aged population.

## 4 Experimental Section

### Cells

293T cells and Vero E6 cells were purchased from the American Type Culture Collection (ATCC, Manassas, VA). The identities of the cell lines were confirmed using STR analysis, and the absence of mycoplasma was confirmed. PBMCs were isolated from the whole blood of healthy donors with Ficoll. CD4^+^ T and CD8^+^ T cells were separated using Miltenyi kits according to the manufacturer's instructions. The cells were maintained in DMEM or RPMI 1640 medium supplemented with 10% fetal bovine serum. The vitamins and cell culture media were purchased from Life Technologies, Inc.

### Sample Collection

All the samples were collected in accordance with a procedure authorized by the Review Board of the Second Military Medical University. Additionally, written informed consent was acquired from each donor. The severity of the disease was determined to be either moderate or severe using a modified version of the WHO interim advice titled “Clinical management of severe acute respiratory infection when COVID‐19 is suspected” (WHO Reference Number: WHO/2019‐nCoV/clinical/2020.4). Mild disease refers to an uncomplicated upper respiratory tract infection (URI) with potential nonspecific symptoms, such as fatigue, fever, cough with or without sputum production, anorexia, malaise, myalgia, sore throat, dyspnoea, nasal congestion, headache, and rarely diarrhea, nausea, and vomiting. This type of disease does not require hospitalization. Severe illness was characterized as a severe infection in the lower respiratory tract or pneumonia accompanied by fever and at least one of the following symptoms: rapid breathing (respiratory rate over 30 breaths per minute), difficulty breathing, or oxygen saturation below 93% when breathing normal air. Table S1 (Supporting Information) presents a comprehensive summary of the attributes of the donors. Serum was collected in 10‐mL vials without anticoagulant. The collected serum was then centrifuged at 2500 rpm for 15 min. After centrifugation, the serum was divided into smaller portions and stored at −20 °C for future research. Peripheral blood mononuclear cells (PBMCs) were obtained from blood samples collected in K3EDTA or lithium heparin tubes using density gradient centrifugation. Briefly, blood was placed on a density gradient (Lymphoprep, STEMCELL Technologies), and PBMCs were isolated via centrifugation at 2000 rpm for 30 min. The PBMCs were then washed four times with phosphate‐buffered saline and frozen in liquid nitrogen using a solution consisting of 90% fetal bovine serum (FBS) and 10% DMSO (Honeywell) until they were used in the stimulation assays.

### SARS‐CoV‐2 S ELISA

The SARS‐CoV‐2 Omicron S protein was obtained from Sino Biological, Inc. (Beijing, China). Corning 96‐well half‐area plates (Thermo Fisher 3690) were covered with a layer of 1 mg mL^−1^ SARS‐CoV‐2 S and incubated overnight at 4 °C. The ELISA methodology was typically implemented in accordance with prior reports.^[^ ^45^ ^]^ The plates were blocked the next day with 3% milk (by weight/volume, skim milk powder, Thermo Fisher, LP0031) in phosphate‐buffered saline (PBS) supplemented with 0.05% Tween‐20. The wells were blocked for 2 h at room temperature. Plasma was subsequently introduced onto the plates, which were subsequently incubated for 1.5 h at ambient temperature. Before adding plasma to the plates, the plasma was subjected to heat inactivation at 56 °C for 30–60 min. The plasma was diluted in a solution of 1% milk and 0.05% PBS‐Tween 20. The dilution process started with a 1:3 ratio, and each subsequent sample was diluted by a factor of 3. The wells were subsequently rinsed five times with a 0.05% PBS‐Tween 20 solution. The secondary antibodies were diluted in a mixture containing 1% milk and 0.05% Tween‐20 and then incubated for 1 h. A 1:5000 dilution of a goat antihuman IgG peroxidase antibody (Sigma A6029) was utilized to detect IgG. The IgM assay utilized a 1:10 000 dilution of a goat antihuman IgM peroxidase antibody (Sigma A6907). The IgA analysis utilized Hybridoma Reagent Laboratory HP6123‐HRP, which is an antihuman IgA horseradish peroxidase antibody. It was employed at a dilution of 1:1000. The wells were rinsed five times with a 0.05% PBS‐Tween 20 solution. The wells were treated with reagents from the TMB Substrate Kit (Thermo Scientific 34021) for 15 min at room temperature. The process was halted using a 2 m solution of sulfuric acid. The plates were analyzed using a SpectraMax plate reader at a wavelength of 450 nm and SoftMax Pro software. The optical densities (ODs) were then adjusted by subtracting the background. A positive control standard was generated by combining plasma samples from six patients who were recovering from COVID‐19. A positive control was included in each plate and utilized to determine the titres (expressed in relative units) of all samples via nonlinear regression interpolations. This method was employed to measure the levels of anti‐S IgG, anti‐S IgM, and anti‐S IgA in each sample. Titers were plotted for each sample and compared to those of samples that tested negative for COVID‐19. As an additional analytical method, the area under the curve was computed for each sample to compare COVID‐19 samples with negative samples. A baseline value of 0.05 was used for peak calculations.

### Neutralizing Antibody Assays

The SARS‐CoV‐2 pseudovirus was constructed using published methods.^[^ ^46^ ^]^ The SARS‐CoV‐2 Omicron spike gene was chemically synthesized, cloned, and inserted into a eukaryotic expression plasmid. 293T cells were first transfected with the S expression vector and then infected with a VSV pseudotyped virus (G∗Δ*G*‐VSV), in which the VSV‐G gene was substituted with luciferase expression cassettes. The culture supernatants were harvested and filtered at 24 h postinfection. The SARS‐CoV‐2 pseudovirus could not be neutralized with anti‐VSV‐G antibodies, and no G∗ΔG‐VSV was mixed with the SARS‐CoV‐2 pseudovirus stock.

Pretitrated amounts of SARS‐CoV‐2 pseudovirus were incubated with serially diluted human sera or plasma at 37 °C for 1 h before being added to confluent Vero cell monolayers in 96‐well plates. The infection process lasted for 12–16 h at 37 °C in an environment containing 5% CO_2._ Afterward, the cells were treated with a fixative solution containing 4% paraformaldehyde and stained with 1 microgram per milliliter of Hoechst dye. The cells were visualized, and the infection was measured by automatically counting the total number of cells and the number of cells that expressed GFP. The infection rate was standardized by comparing the number of cells infected with SARS‐CoV‐2 pseudovirus when incubated with normal human serum to the average value. The limit of detection (LOD) was determined to be less than 1:20. The data are displayed as the relative infection rate for each concentration of serum. The IC50 titres for neutralization were determined using GraphPad Prism 8.0.

### Stimulations for the Detection of SARS‐CoV‐2‐Specific CD4^+^ and CD8^+^ T Cells

The SARS‐CoV‐2 peptides were produced as raw materials by Genomeditech (Shanghai, China). A set of 15‐mer peptides, with a 10 amino acid overlap, was produced to span the whole length of the S protein from the Omicron (BA.5) variant. Each peptide was separately dissolved in DMSO at a dosage of 10–20 mg mL^−1^. Megapools were created by combining small amounts of these individual peptides, followed by a freeze‒drying process, and then reconstituting them in DMSO at a concentration of 1 mg mL^−1^. PBMCs were thawed in RPMI 1640 medium (Gibco) containing 10% human serum (Sanquin, Rotterdam), penicillin (100 IU mL^−1^; Lonza, Belgium), streptomycin (100 µg mL^−1^; Lonza, Belgium), and 2 mm L‐glutamine (Lonza, Belgium; R10H medium). The cells were then exposed to benzonase (50 IU mL^−1^; Merck) at 37 °C for 30 min. Next, one million peripheral blood mononuclear cells (PBMCs) were exposed to SARS‐CoV‐2 variant peptide pools at a concentration of 1 µg mL^−1^ per peptide in 200 µL of solution. This step was performed in a 96‐well U‐bottom plate, and cells were incubated at 37 °C for 20 h. The cells were also treated with an equimolar dose of DMSO (negative control) or a combination of phorbol 12‐myristate 13‐acetate (50 µg mL^−1^) and ionomycin (500 µg mL^−1^) (positive control). Furthermore, peripheral blood mononuclear cells (PBMCs) were activated using a peptide pool containing 176 well‐known peptides that target various HLA subtypes and different infectious agents, such as human herpesviruses and influenza virus. This peptide pool, known as the PepMix CEFX Ultra SuperStim Pool provided by JPT, served as a positive control for the experiment. Following stimulation, the cells were subjected to staining to identify markers of different lymphocytes.

### Generation of Antibodies

The genes encoding the ectodomain of the S trimer from the prototypic SARS‐CoV‐2 Wuhan‐Hu‐1 strain (GenBank: MN908947.3) were constructed in accordance with previous reports.^[^ ^47^ ^]^ As previously reported, S3H3, CV3‐25, ACE‐Fc, and humanized UCHT1 (anti‐CD3; GenBank accession numbers AAB24133.1 and AAB24132.1) antibodies were prepared. The bispecific CrossMabs SS12, SC02, and SA07 were generated from S3H3/UCHT1, CV3‐25/UCHT1, and ACE2‐Fc/UCHT1 using the “knobs into holes” (KiH) and CrossMab methodologies, respectively. The heavy and light chains of various antibodies were cloned into the pcDNA3.1 (+) expression vector using the Freestyle 293 expression system (Invitrogen), as previously described.^[^ ^48^ ^]^ Using affinity chromatography, recombinant antibodies were isolated from the serum‐free culture supernatant. The absorbance was measured at 280 nm to calculate protein concentrations, and SDS‒PAGE analysis was used to confirm purity. Using antibody affinity assays, bioactivity was measured.

### Affinity Measurement

Previously described surface plasmon resonance (SPR) measurements with a BIAcore 2000 instrument were performed to determine the binding affinities of the IgGs.^[^ ^29^, ^48^ ^]^ Using standard amine‐coupling chemistry, antihuman Fc polyclonal antibodies (Jackson ImmunoResearch Europe Ltd.) were immobilized on a CM5 chip [at 150 relative units (RUs)]. Following capture of the antibodies, recombinant proteins were injected into the flow cell. This study utilized the CD3 epsilon‒delta heterodimeric protein (Acro Biosystems) and spike trimer protein. The binding response was corrected by subtracting the RUs from a blank flow cell. the 1:1 Langmuir model of simultaneous fitting of ka and kb for the kinetic analysis was used.

### Pharmacokinetics

IgGs were injected intravenously into the tail veins of female C.B‐17 SCID mice aged 7 weeks. Retroorbital bleeding was performed to collect blood samples daily from Day 1 to Day 15 in tubes coated with heparin to prevent clotting. Six mice were required at each time point, and each mouse was bled only once. After the removal of the cells via centrifugation, the plasma samples were frozen at –80 °C until analysis. Antibody concentrations in the serum were measured using a competitive ELISA. Briefly, serum samples were incubated with a subsaturating concentration of the indicated biotin‐conjugated antibodies on target protein‐coated ELISA plates for 1 h at 37 °C. Detection was performed using avidin conjugated with alkaline phosphatase. A noncompartmental analysis was used to calculate the PK parameters.

### In Vitro Cell Killing and T‐Cell ACTIVATION ASSAYS

For S^+^ cell killing assays, live S^+^ cells were gated as PI‐S^+^ 293 cells by FACS, and the absolute cell count was obtained by adding fluorescein isothiocyanate beads as an internal counting control to the reaction mixture. CD69 and CD25 surface expression facilitated the detection of activated T cells. The induction of intracellular granzyme B expression was detected using FACS. The perforin concentration in the medium was determined using ELISA (eBioscience). Each antibody was obtained from BD Biosciences.

### In Vitro Transmission Assay

As previously reported,^[^ ^15^ ^]^ cell‐to‐cell and cell‐free pseudotyped viruses were established. Briefly, in the lentiviral vector system, the expression of the antisense reporter gene Gluc is hindered by an intron oriented in the sense direction of the HIV‐1 genome, and thus Gluc production occurs only in infected target cells and not in virus‐producing cells.^[^ ^15^, ^49^ ^]^ By coculturing the virus‐producing and target cells and measuring the Gluc activity in the media from the coculture between donor cells (such as 293T) producing lentiviral pseudotypes and target cells (such as 293T/ACE2), cell‐to‐cell transmission was determined. The HIV‐1 NL4.3‐inGluc reporter was constructed as previously reported^[^ ^49^ ^]^ using the NL4‐3 plasmid (Addgene 44965), and pcDNA3.1‐SARS‐CoV‐2‐S encoding the full‐length spike protein was constructed in a previous study.^[^ ^26^ ^]^ 293T cells were specifically seeded in 6‐well plates and transfected with 2 µg of NL4.3‐inGluc and 1 µg of plasmids encoding the SARS‐CoV‐2 spike protein. The following day, transfected 293T donor cells were incubated with PBS/5 mm ethylenediaminetetraacetic acid (EDTA) and thoroughly washed with PBS to remove the EDTA, followed by coculture with the target cells at a 1:1 ratio and PBMCs (1:10 cell ratio to 293T/ACE2 cells) in 12‐well plates for 24–72 h. As needed, therapeutic IgGs were added. Gluc activity in the supernatants was determined. In addition to cell‐to‐cell transmission, cell‐free infection was performed in the Transwell system in this study. Briefly, an equal number of transfected donor cells were seeded onto the insert, while target cells, which were again mixed with an equal number of untransfected 293T cells, were seeded on the bottom with PBMCs. This approach prevented donor and target cells from being in contact while allowing the virus to spread through the filter. Gluc activity was measured in supernatants collected at the same time points used for cell‐to‐cell transmission assays.

### In Vivo Experiments

In vivo experiments were approved by the Institutional Animal Care and Use Committee (IACUC) of Second Military Medical University and were performed in animal biosafety level 3 (BSL‐3) containment in compliance with institutional and federal guidelines. The preparation and susceptibility of the ACE2 humanized (AVV‐hACE2) mouse model to SARS‐CoV‐2 infection were previously described.^[^ ^32^ ^]^ The authentic wild‐type SARS‐CoV‐2 and SARS‐CoV‐2 Omicron BA.5 strains were obtained from the Zhejiang and Guangdong Provincial Center for Disease Control and Prevention, China. At least 7 days after the application of the AAV‐hACE2 virus particles, 12‐month‐old human CD3E knock‐in C57BL/6N female mice were administered 5 × 10^9^ viral genome copies in 40 µL of PBS via forced inhalation. Ketamine and xylazine were injected intraperitoneally into the mice to induce anesthesia (0.1 mg g^−1^ body weight and 0.01 mg g^−1^, respectively). Every effort was made to reduce the suffering of the animals. The mice were observed and weighed daily over a period of 8 days for prophylaxis studies and 15 days for treatment studies. The mice were intraperitoneally injected with various therapeutic IgGs either 24 h prior to infection or 3 days after infection. The mice were euthanized at the indicated times after infection or when they exhibited clinical signs of severe disease, including any of the following: marked lethargy or inactivity, severe respiratory distress or labored breathing, an inability to ambulate, or weight loss of more than 30% from the baseline.

### Quantification of Serum IgG Levels

Blood was collected in microvette serum gel tubes and fractionated by centrifugation (10 000 × g, 5 min). ELISA was used to measure IgG levels in accordance with previously described protocols.^[^ ^50^ ^]^

### Measurement of the Viral Burden

Tissues were weighed, lysed in TRIzol (Thermo Fisher), and dissociated using a gentle MACS Octo Dissociator in gentle MACS M tubes (Miltenyi Biotec). The samples were transferred to Phasemaker tubes (Thermo Fisher), and 0.2 mL of chloroform per milliliter of TRIzol was added. After vigorously shaking the tubes for 5 min, they were centrifuged for 15 min at 12 000 × g and 4 °C. The RNA‐containing aqueous phase was transferred to a new tube, and RNA was extracted using an RNeasy Mini Kit (Qiagen). The TaqMan RNA‐to‐CT 1‐Step Kit was used to reverse transcribe and amplify the RNA (Thermo‐Fisher). Reverse transcription was conducted at 48 °C for 15 min, followed by 2 min at 95 °C. Amplification was accomplished over 50 cycles as follows: 95 °C for 15 s and 60 °C for 1 min. The number of RNA copies of the SARS‐CoV‐2 N gene was determined in the samples using a previously published assay.^[^ ^51^ ^]^ Briefly, a TaqMan assay targeting a highly conserved region of the N gene was developed (forward primer: ATGCTGCAATCGTGCTACAA; reverse primer: GACTGCCGCCTCTGCTC; probe: /56‐FAM/TCAAGGAAC/ZEN/AACATTGCCAA/3IABkFQ/). This region was incorporated into an RNA standard to enable the determination of copy numbers at as low as 10 per reaction. The final concentrations of the primers and probe in the reaction mixture were 500 and 100 nm, respectively.

### Lung Histology

The lungs of euthanized mice were instilled with 10% neutral buffered formalin and then submerged in 10% formalin overnight for fixation. The tissues were fixed, embedded in paraffin, sectioned at a thickness of 4 µm, and stained with hematoxylin and eosin. A board‐certified veterinary anatomic pathologist evaluated the lung sections microscopically, and representative images were captured.

### Statistical Analysis

Unless stated otherwise, Student's *t*‐test was used to determine the significance of differences between 2 groups, and analysis of variance (ANOVA) was used to determine the significance of differences between 3 or more groups. When *p* < 0.05, differences between samples were regarded as statistically significant.

## Conflict of Interest

J.Z. is a shareholder at KOCHKOR Biotech Inc., Shanghai and W.F. and S.H. are inventors on intellectual property related to this work. The other authors declare no competing interests.

## Supporting information



Supporting Information

## Data Availability

The data that support the findings of this study are available from the corresponding author upon reasonable request.
