# Supplementary material for: T‐Cell‐Dependent Bispecific IgGs Protect Aged Mice From Lethal SARS‐CoV‐2 Infection
Source: Adv Sci (Weinh). 2025 Feb 20;12(15):2406980. doi: 10.1002/advs.202406980 (PMC12005765; doi:10.1002/advs.202406980)
Supplement: Supplementary file 1 — Supporting Information [file ADVS-12-2406980-s001.pdf]

ADVANCED SCIENCE
Open Access

Supporting Information

for *Adv. Sci.*, DOI 10.1002/advs.202406980

T-Cell-Dependent Bispecific IgGs Protect Aged Mice From Lethal SARS-CoV-2 Infection

*Wenyan Fu, Wei Zhang, Zhongshuai You, Guangyao Li, Chuqi Wang, Changhai Lei, Jian Zhao, Jin Hou and Shi Hu**

Supplementary Figures

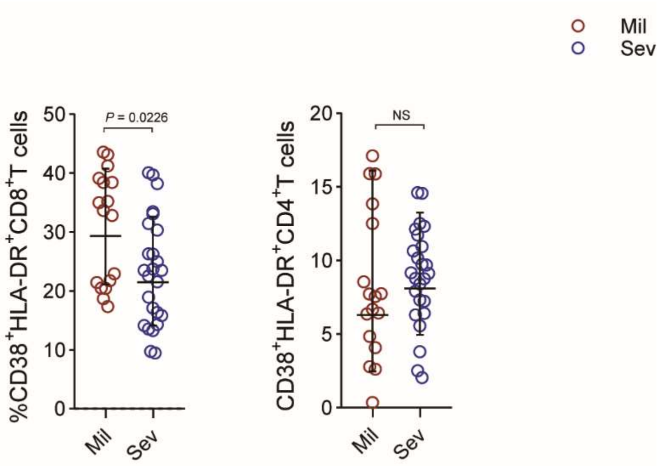

Figure S1. Relationships between T cell activation and COVID-19 severity. CD8+ T cells or CD4+ T cells in different SARS-COV-2 infected patient groups was assessed Data are expressed as mean ± SD. P values are from two-sided nonparametric t tests.

a

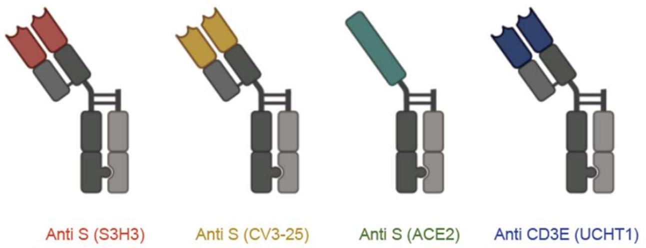

b

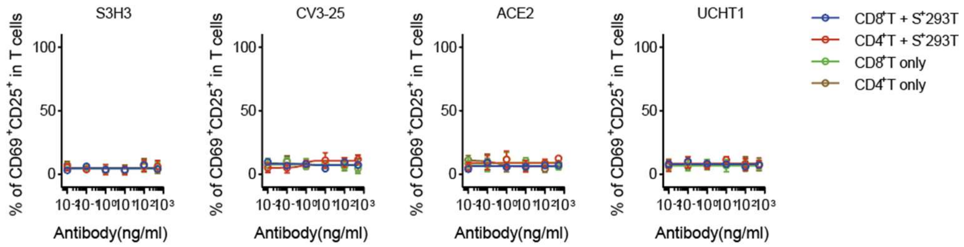

Figure S2. Single-armed antibodies are insufficient to activate T cells. a. Schematic representation of single armed antibodies. b. 293/S cells and purified $CD8^{+}$ T cells or $CD4^{+}$ T cells, or $CD8^{+}$ T cells alone were incubated with various concentrations of antiboies for 24 hours. The T-cell–to–293 cell ratio was 5:1. Cell killing and activated T cells, marked as $CD69^{+}CD25^{+}$ cells, were measured and calculated.

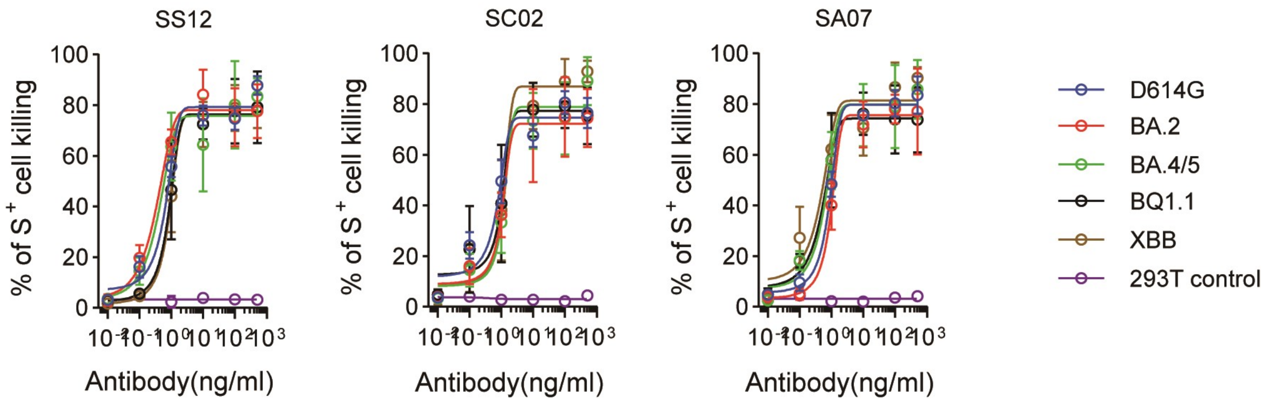

Figure S3. Anti-S/CD3 TDB activates T cells in the presence of the target cell with S protein derived from SARS-CoV-2 VOCs. Different 293/S cells and PBMCs isolated from healthy donors (1:10 cell ratio) were incubated with various concentrations of S-TDBs or F(ab′)2 S-TDB for 24 hours (data shown as the means ± SDs, n = 6).

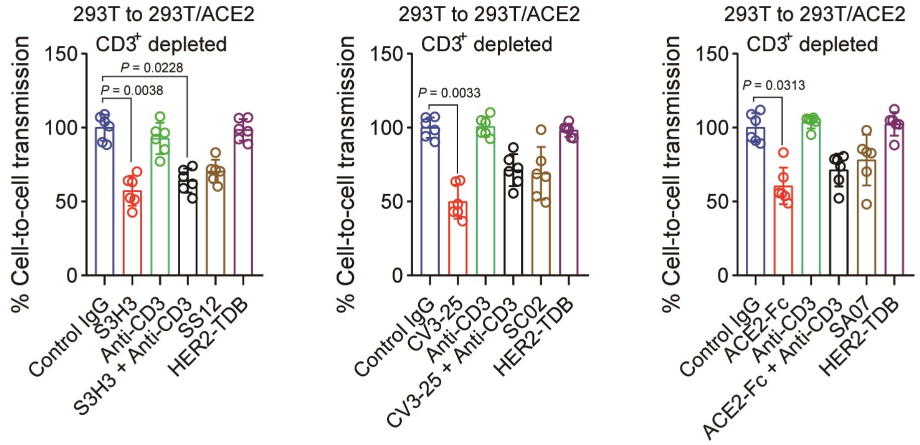

Figure S4. T cells are necessary for the efficient suppression of cell-to-cell infection by S-TDBs. Comparison of cell-to-cell transmission mediated by SARS-CoV-2 indicated that therapeutic IgGs were present with PBMCs depleted of CD3+ T cells during the infection period. Results shown were from six independent experiments, with cell-free infection measured at 48 and 72 h after coculture. Data are mean ± s.d., *P* values are from one-way ANOVA followed by Tukey's post-test.

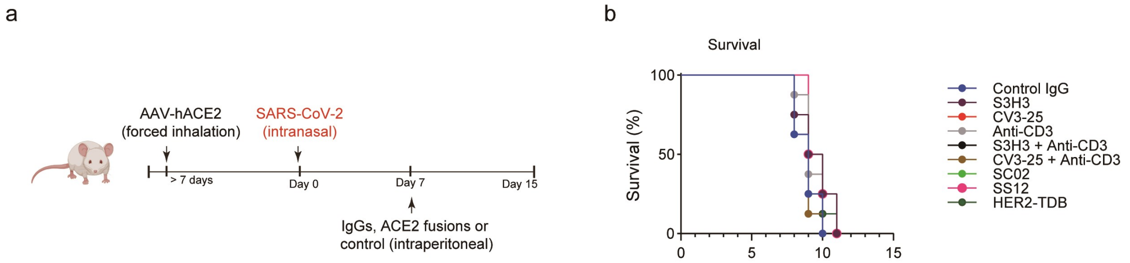

Figure S5. S-TDB treatment against SARS-CoV-2 in aged mice. a. Humanized CD3e mice were transduced with AAV-hACE2 by forced inhalation. After >7 days, the mice were inoculated intraperitoneally with different S-TDBs or control Abs (12 mg/kg for each drug) 7 days (black arrow) after being infected intranasally with SARS-CoV-2. **B.** Survival analysis (n = 8).

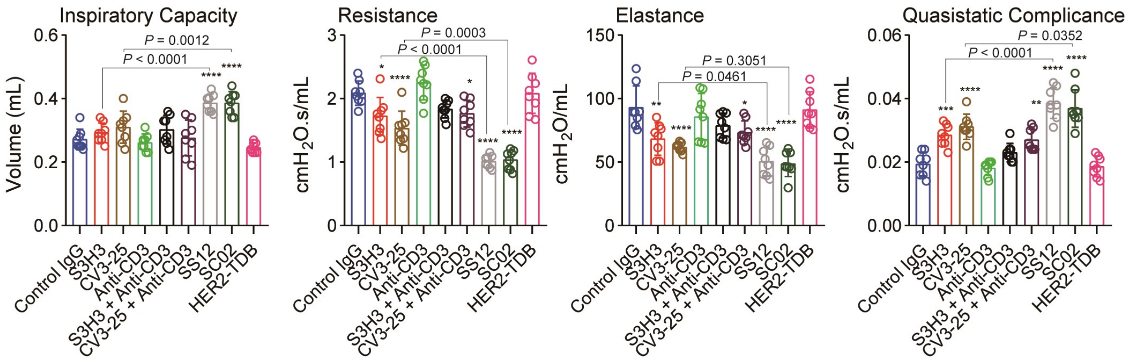

Figure S6. S-TDB treatments improved pulmonary mechanics. g. Parameters of respiratory mechanics: inspiratory capacity, resistance, elastance, and quasistatic compliance measured at 8 dpi (n = 7 or 8). Data are mean ± s.d., *P* values are from one-way ANOVA followed by Tukey's post-test, * $P < 0.05$; ** $P < 0.01$, *** $P < 0.001$, **** $P < 0.0001$; compared with Control IgG .

Supplementary Tables

Table S1. Participant characteristics.

	Mild & Moderate (8 & 9)	Severe & Critical (22 & 3)
**Age (years)**	67-84 [Median = 79, IQR =9]	65-83 [Median = 72, IQR =7]
**Gender**		
Male (%)	82.4% (12/17)	91% (22/25)
Female (%)	17.6% (3/17)	8% (2/25)
**Vaccination**		
Unvaccinated (%)	11.8% (2/17)	4%(1/25)
Partially vaccinated (%)	5.88% (1/17)	0% (0/25)
Full vaccination (%)	41.18% (7/17)	44% (11/25)
Booster (%)	41.18% (7/17)	52% (13/25)

**Hospitalization status**		
Never hospitalized (%)	(4/17)	(0/25)
Hospitalized at the time of blood collection (%)	(11/17)	(25/25)
**SARS-CoV-2 PCR Positivity**	100% (17/17), 17 tested	100% (25/25), 25 tested
**Disease Severity at Time of Blood Collection**		
Healthy/No disease (0)	(0/17)	(0/25)
Mild (1-2)	(8/17)	(0/25)
Moderate (3-5)	(9/17)	(0/25)
Severe (6-7)	(0/17)	(22/25)
Critical (8-9)	(0/17)	(3/25)
Fatal (10)	(0/17)	(0/25)
**Presenting Symptoms**		
Cough (%)	14	18

Fatigue (%)	11	19
Fever (%)	11	20
Anosmia (%)	6	22
Dyspnea (%)	9	11
Diarrhea (%)	3	12
**Days Post Symptom Onset at Collection**	5-33 [Median = 13,IQR = 14]	4-31 [Median = 16,IQR = 18]
**Past Medical History**		
No known (%)	4	6
Hyperlipidemia (%)	3	5
Hypertension (%)	5	5
Coronary artery disease (%)	1	1
Diabetes (%)	2	9
Obesity (%)	**3**	8

Other (%)	5	11
**Known or suspected sick contact/exposure (%)**	14	25

Table S2. COVID-19 disease severity classifications

Disease Severity Score (0-10)	Description	Hospitalization Requirement	Supplemental Oxygen Needs for COVID-19
Healthy			
0	No known COVID-19 diagnosis or convalescent (fully recovered from COVID-19 and 3 weeks or more from initial diagnosis, without symptoms)	No	None
Mild			
1	Subclinical infection or asymptomatic (no symptoms preceding or up to time of COVID-19 diagnosis)	No	
2	Symptoms consistent with COVID-19 without limitation of activities	No	
Moderate			
3	Symptoms consistent with COVID-19 that limit activities and/or with home oxygen requirement (above baseline)	No	+/- home supplemental oxygen (above baseline)
4	Hospitalized for a reason other than COVID-19 but incidentally found to be positive on testing or recovered from COVID-19 to the extent that hospital care is no longer required for COVID-19 but hospitalization extended for infection-control or other reasons unrelated to COVID-19 management	Yes, but not requiring (ongoing) hospital care for COVID-19	None

5	Requiring ongoing medical care for COVID-19 other than supplemental oxygen (non-ICU level care)	Yes (non-ICU)	None
Severe			
6	Requiring ongoing medical care for COVID-19 including supplemental oxygen via nasal cannula	Yes (non-ICU)	Nasal cannula
7	Requiring ongoing medical care for COVID-19 including high-flow supplemental oxygen or noninvasive ventilation	Yes (non-ICU)	High-flow oxygen delivery device or non-invasive ventilation
Critical			
8	Requiring vasopressor support or other ICU level care for COVID-19 but not mechanical ventilation or extracorporeal membrane oxygenation (ECMO)	Yes (ICU)	+/- but not requiring mechanical ventilation or ECMO
9	Requiring ICU level care including mechanical ventilation or ECMO for management of COVID-19	Yes (ICU)	Mechanical ventilation or ECMO
Fatal			
10	Death attributable to COVID-19	+/-	+/-

*Quiescent storage for 4 week, 40 °C, 1 mg/mL

**Affinity KD analyzed using a BIAcore T100. The data were globally analyzed using a simultaneous fit for both dissociation (kd) and association (ka).

The value for KD was calculated as kd/ka (nanomolar, nM).

Table S3. Selected analytical and affinity data of antibodies.

Parameter	Unit	S3H3	CV3-25	ACE2-Fc	UCHT1	SS12	SC02	SA07
HMW formation after storage*	% SEC area	< 0.1	< 0.1	< 0.1	< 0.1	< 0.1	< 0.1	< 0.1
LMW formation after storage*	% SEC area	< 0.1	< 0.1	< 0.1	< 0.1	< 0.1	< 0.1	< 0.1
Affinity/kinetics of S binding** (SPR)	Ka (1/Ms)	1.57×10^5	6.08×10^5	2.63×10^5	-	7.75×10^5	9.04×10^5	8.89×10^5
	Kd (1/s)	9.02×10^{-4}	1.43×10^{-3}	2.54×10^{-3}	-	4.8×10^{-3}	4.36×10^{-3}	8.35×10^{-3}
	KD (nM)	5.75	2.35	10.76	-	6.20	4.82	9.39
Affinity/kinetics of CD3delta-epsilon binding	Ka (1/Ms)			6.71×10^5	6.71×10^5	8.93×10^5	9.74×10^5	8.17×10^5
	Kd (1/s)			1.05×10^{-4}	1.05×10^{-4}	2.8×10^{-4}	2.85×10^{-4}	3.02×10^{-4}
	KD (nM)			0.16	0.16	0.31	0.29	0.37

*Quiescent storage for 4 week, 40 °C, 1 mg/mL

**Affinity KD analyzed using a BIAcore T100. The data were globally analyzed using a simultaneous fit for both dissociation (kd) and association (ka). The value for KD was calculated as kd/ka (nanomolar, nM).

Table S4 Pharmacokinetic parameters of recombinant antibodies in mice.

	S3H3-biotin-based competitive ELISA		CV3-25-biotin-based competitive ELISA		ACE2-Fc -biotin-based competitive ELISA	
Parameter	S3H3	SS12	CV3-25	SC02	ACE2-Fc	SA07
AUC (day µg ml-1)	533.16	485.97	551.39	456.16	497.6	445.20
T1/2 (day)	9.53	10.13	8.27	9.03	11.16	9.34
CL (ml day-1 kg-1)	6.09	6.60	6.09	7.23	6.04	7.24
VSS (ml kg-1)	85.38	94.71	78.55	96.90	96.22	99.84

Pharmacokinetic parameters were calculated using a noncompartmental analysis. AUC, area under the concentration versus time curve; t1/2, half-life; CL, clearance; VSS, steady-state volume of distribution.
